# Ischemic Stroke as a Form of Presentation of Aortic Dissection: A Case Report

**DOI:** 10.7759/cureus.52866

**Published:** 2024-01-24

**Authors:** Noélia Carrillo-Alfonso, Marta Mugeiro, Inês Amado, Ernesto Ruivo, Ana Lares

**Affiliations:** 1 Anesthesiology, Centro Hospitalar Universitário do Algarve, Faro, PRT

**Keywords:** prehospital management, prehospital emergency medicine, emergency service, ischemic stroke, atypical presentation of aortic dissection

## Abstract

Aortic dissection is a rare condition with a high mortality rate, the clinical manifestations of which depend on the involvement of aortic branches. We describe the case of a male patient with neurological deficits accompanied by dorsal-lumbar pain and exuberant sympathetic hyperactivity, diagnosed with ascending and descending aorta dissection, initially managed in the prehospital setting. This case reinforces the importance of maintaining high levels of suspicion for rare causes of cerebrovascular accidents.

## Introduction

Aortic dissection is an uncommon condition characterized by a high mortality rate, as Castrillo et al. presented in their report [1,2]. It manifests with diverse clinical presentations contingent upon the affected aortic branches [1]. This case report details the activation of the prehospital emergency team for a male patient with ascending and descending aorta dissection, involving the extracranial segment of the right carotid artery. The dissection led to an ischemic stroke accompanied by intense dorsal-lumbar pain and notable signs of sympathetic hyperactivity, as in the case reported by Lee et al. [2]. By presenting this case, we emphasize the importance of maintaining a heightened suspicion for uncommon etiologies in the context of cerebrovascular accidents. 

## Case presentation

The prehospital emergency team was activated for a 68-year-old male presenting with intense sudden-onset dorso-lumbar pain in the last hour. Upon the emergency team's arrival, 20 minutes later, the patient was found lying on the ground, agitated, pale, sweaty, nauseated, and with marked flushing on the left hemiface and pallor on the right hemiface. Further physical examination revealed no adventitious sounds on auscultation and capillary refill time in the left upper limb <2 seconds; neurological examination showed Glasgow Coma Scale 14/15, dysarthria, left-sided facial droop, and left hemiplegia. Capillary refill time in the right upper limb was >3 seconds. The patient's medical history revealed hepatocellular carcinoma treated surgically in 2019, an ischemic stroke without sequelae in 2013, and a previously controlled arterial hypertension, with the patient currently on lercanidipine 10 mg and aspirin 100 mg as regular medications. 

Vital signs assessment showed difficulty in obtaining oximetry readings from the right upper limb, with a peripheral O2 saturation measurement of 98% in the left upper limb in ambient air, a heart rate of 58 bpm, and blood pressure of 205/82 mmHg in the left upper limb. An electrocardiogram indicated sinus rhythm with no criteria for acute ischemia. The hypothesis of ischemic stroke with activation of the Stroke Fast Track was communicated to the Urgent Patient Guidance Center. Ondansetron 4 mg and fentanyl 0.05 mg IV (intravenous) were administered for nausea control and analgesia, respectively.

Due to persistent intense dorso-lumbar pain with signs of sympathetic hyperactivity and a noticeable difference between the pallor of the right hemiface and the flushing of the left hemiface, a suspicion of alterations in the arterial vascularization of these territories due to aortic dissection was raised. Secondary assessment revealed asymmetry of radial pulses, with a decrease in the right, blood pressure of 153/63 mmHg in the left upper limb and 126/86 mmHg in the right upper limb, and palpable and symmetric femoral pulses. After effective analgesia with fentanyl 0.075 mg, updated information was transmitted to the Urgent Patient Guidance Center, informing them of the diagnostic hypothesis of ischemic stroke due to ascending aortic dissection.

The patient was taken to the Emergency Department with minimal abrupt movements and sudden speed changes. The patient remained hemodynamically stable during transportation, maintaining a systolic blood pressure differential of more than 20 mmHg in both arms. The patient arrived one hour and 30 minutes after the prehospital emergency team activation. In the Emergency Room, a detailed neurological examination revealed drowsiness but arousable to verbal stimulation, dysarthria, major left central facial paresis, left anisocoria, rightward gaze preference, left hemiplegia, and left hemihyposthesia, with extensor plantar reflex on the left. The National Institutes of Health Stroke Scale (NIHSS) score was 15/42.

**Figure 1:**
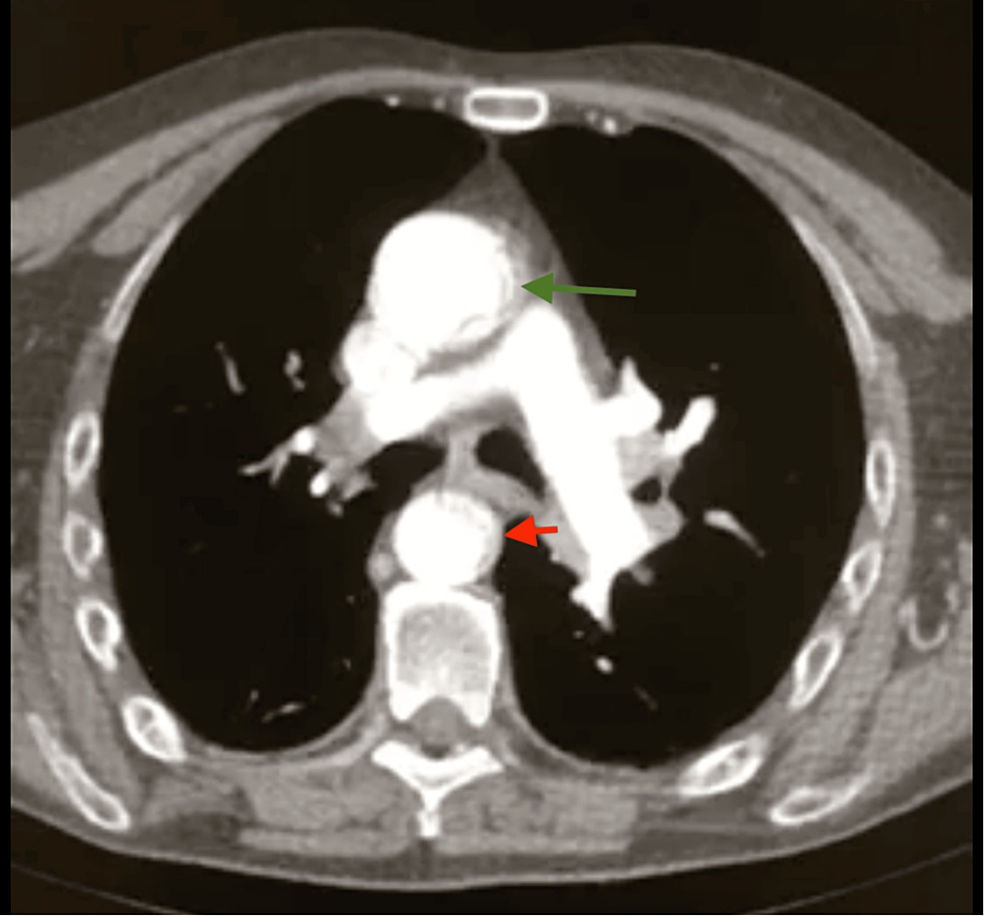
Thorax CT angiography demonstrating aortic dissection of the ascending aorta (green arrow) and descending aorta (red arrow), showing true and false lumen opacified with contrast

**Figure 2:**
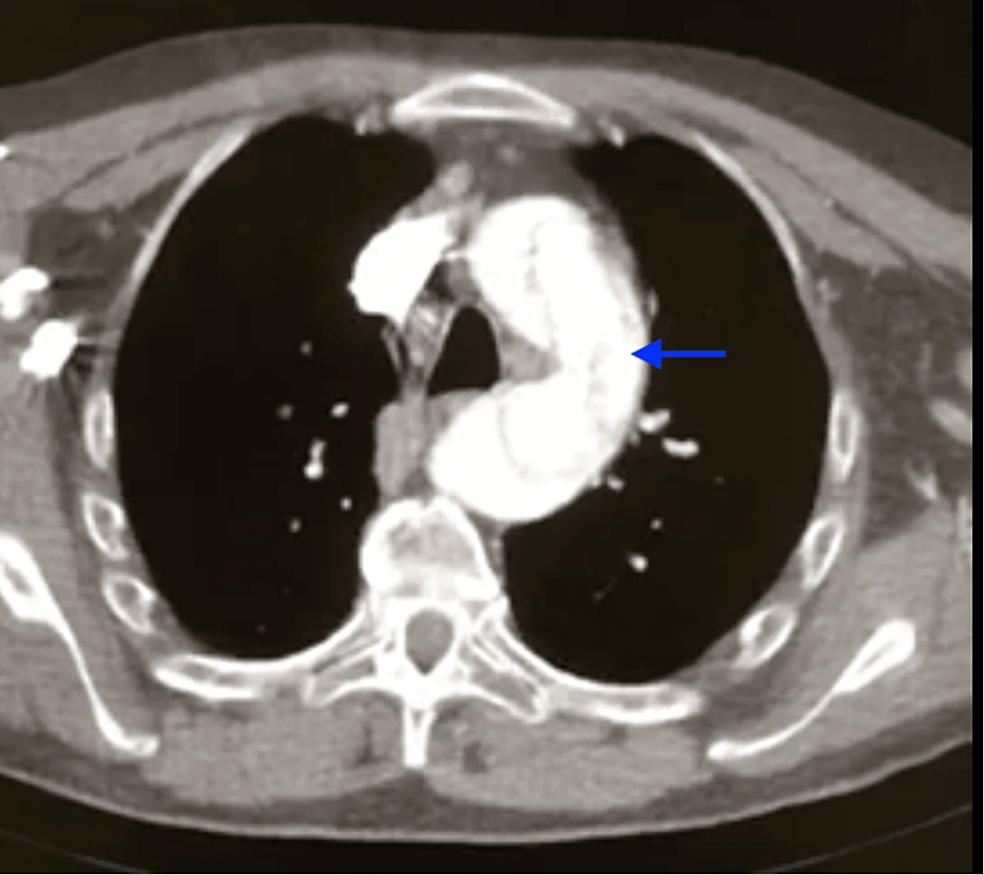
CT angiography showing aortic dissection at the level of the aortic arch; blue arrow points to the false lumen

A contrast-enhanced CT scan directed at the neck vessels was performed (Figures 1, 2). It reported a type A aortic dissection according to the Stanford classification, extending from the aortic root to the level of the abdominal aorta above the emergence of the renal arteries. It did not present a visible progression of the dissection to the supra-aortic vessels or major abdominal branches, but it didn't show opacification with contrast of the right common carotid artery. Both true and false lumens showed opacification with contrast.

A final diagnosis was made of ascending and descending aortic dissection, with right extracranial carotid extension. At this point, five hours had elapsed since the onset of symptoms. Since the hospital lacked cardiothoracic and vascular surgery services, the case was discussed with the referral center (three hours away by helicopter).

A conservative approach with medical treatment was chosen due to the patient's high pre-operative neurological disability (NIHSS 15), the calculated time until arrival at the surgical center (at least eight to nine hours until surgery), and the risk of hemorrhagic transformation of brain infarction. This decision was made while acknowledging the high surgical risk and mortality rate associated with the intervention, as well as the elevated mortality risk linked to medical treatment [3,4].

Taking the above into account, labetalol and opioid perfusion were chosen for blood pressure and symptomatic control, respectively. The family was informed of the guarded prognosis and the decision not to resuscitate in case of cardiac arrest. The clinical case progressed unfavorably with dissection of the abdominal aorta and renal arteries, acute kidney injury with anuria, and hemorrhagic dyscrasia. The patient succumbed to the condition 36 hours after the onset of symptoms.

## Discussion

Aortic dissection is a rare pathology associated with a high mortality rate. It is more common in males in their seventh decade of life and with a personal history of hypertension [1]. Other risk factors include smoking, obesity, and connective tissue diseases, and there is an agreement among many risk factors for this pathology and ischemic stroke [1,2,5].

Clinical presentations of aortic dissection vary widely, contingent upon the specific aortic branches involved. Typically, the onset manifests as severe and abrupt chest, abdominal, or interscapular pain [1,2]. Nevertheless, under specific conditions, aortic dissection can be an uncommon cause of ischemic stroke, and the onset may be marked by new neurological deficits [1,2,5]. 

Type A acute aortic dissection (AAAD) treated with conservative treatment has a mortality rate of 0.5% per hour (23.7% at 48 hours), decreasing to 4.4% in a surgical group [6]. Poor clinical outcome in AAAD was linked to older age, higher incidence of hypertension, history of stroke, and common carotid artery occlusion [7].

The involvement of carotid branches, leading to cerebral ischemia, necessitates the restoration of cerebral blood flow promptly, avoiding the creation of new brain infarctions or hemorrhages. Aggressive reconstruction of the arch and carotid arteries can enhance neurological outcomes in type A aortic dissection, contingent on rapid transport-to-incision-to-cardiopulmonary bypass [3,4,6-10]. Sasaki et al. proposed an aorto-carotid bypass with immediate central repair for AAAD patients complicated by carotid artery occlusion, demonstrating neurological deficit recovery in 127 patients with 0% hospital mortality [10]. However, these results were associated with a time from symptom onset to the operating room of 7.2 ± 2.4 hours [10]. Tsukube et al. reported a high rate of consciousness recovery in comatose AAAD patients with aortic repair within five hours of symptom onset [3]. Hemorrhagic conversion in the brain was rare with early surgical repair, even with full anticoagulation [3,4].

Scientific literature highlights a strong correlation between the time from onset to surgery and outcomes: an NIHSS score greater than 11 and a period of 9.1 hours between symptom onset and surgery reliably predict a lack of neurological improvement [3,4,10].

In cases where immediate surgical intervention is not possible, such as due to the distance to the cardio-thoracic center, managing AAAD with cerebral ischemia becomes extremely challenging. Medical treatment carries mortality rates as high as 86% [3]. To improve brain circulation, blood pressure should be maintained at higher levels, but this poses risks of aortic rupture, cardiac tamponade, or retrograde dissection with coronary artery involvement. On the other hand, aortic dissection requires lower blood pressure control using drugs like labetalol to alleviate transmural tension and prevent aneurysm rupture, increasing the risk to the brain.

The reported patient had an NIHSS score of 15, with at least eight hours from symptom onset until reaching a surgical center, not including the time for necessary anesthesia and surgical arrangements. Considering cutoff points predicting neurological improvement and the mortality rates associated, medical treatment was proposed for this patient. 

## Conclusions

In the realm of differential diagnoses for ischemic stroke, consideration of less probable causes associated with a heightened mortality rate is imperative. Timely identification is crucial for enhancing patient survival after type A aortic dissection with carotid artery involvement, tipically managed surgically by total arch replacement.

This case demonstrates an atypical presentation of this nosological entity, underscoring the critical importance of maintaining a heightened suspicion for aortic dissection to facilitate a prompt diagnosis. Although, in the present clinical scenario, this did not translate to a favorable outcome, the thorough documentation of this case serves a pivotal role in positioning aortic dissection as a noteworthy consideration in the spectrum of differential diagnoses of an ischemic stroke.
